# Editorial: Ecological interactions between mosquitoes and their microbiota: implications for pathogen transmission

**DOI:** 10.3389/fmicb.2024.1395348

**Published:** 2024-03-28

**Authors:** Mario Garrido, Guillaume Minard, Jesús Veiga, Josué Martínez-de la Puente

**Affiliations:** ^1^Department of Parasitology, University of Granada, Campus Universitario de Cartuja, Granada, Spain; ^2^Univ Lyon, Université Claude Bernard Lyon 1, CNRS, INRAe, VetAgro Sup, UMR Ecologie Microbienne, Villeurbanne, France; ^3^Department of Conservation Biology and Global Change, Doñana Biological Station (EBD, CSIC), Seville, Spain; ^4^Ciber de Epidemiología y Salud Pública (CIBERESP), Madrid, Spain

**Keywords:** mosquito-microbiota interaction, environmental variation, ecological interactions, wildlife, public health, vector-borne diseases, pathogen transmission, holobiont

Vector-borne diseases (VBDs) have spread significantly in recent decades, primarily due to global changes induced by human activities. Mosquitoes stand out as primary vectors of pathogens affecting humans, livestock, and wildlife. Like other vectors, mosquitoes interact with hosts, pathogens, and eventually reservoirs under changing environmental conditions that may affect their biological fitness and, consequently, disease risk. Thereby, environmental factors and human activities at various scales—such as local climate conditions, shifts in land use patterns, or alterations in host community compositions—play pivotal roles in modulating vector competence and, so, the epidemiology of these diseases within natural settings.

Since the onset of the 21st century, the widespread adoption of state-of-the-art molecular techniques as well as manipulation of the mosquito microbiota has significantly advanced our understanding of the pivotal role of the vector microbiota on modulating the development, behavior, digestion, reproduction, physiology, and immunity of its host as well as its ability to replicate and transmit vectored pathogens (Guégan et al., 2018; Gao et al., 2020). Consequently, composition, structure, and dynamics of mosquito associated microbial communities may significantly influence vectorial capacity and the dynamics of mosquito-borne pathogen transmission in the field (Cansado-Utrilla et al., 2021) while it has been poorly used until know to predict the epidemiological risk (Dada et al., 2021). This Research Topic is composed by a literature review and five original research articles addressing different questions on this topic. Cutting-edge publications included in this Research Topic employ both correlational and experimental approaches to identify factors influencing the microbiota of major mosquito species across various scales. The bacterial communities present in mosquitoes differ between developmental stages and even among individuals coexisting in the same environment, even at local scale. Therefore, it is necessary to identify the factors that influence the composition of mosquito microbiota, which may shape the transmission of vector-borne diseases. An example of that could be found in the publication by Yan et al. who study the intricate interplay between seasonality and land cover on *Culex* mosquito abundance and their associated microbial communities, two components that may influence the vectorial capacity. They found that Proteobacteria was the most dominant bacterial phyla in mosquito's microbiome with the intracellular symbiont *Wolbachia* being the most abundant genera. The mosquito microbiota diversity varies between seasons, with a higher microbiome alpha diversity during early-autumn than in late-summer. Furthermore, the relative abundance of *Wolbachia* was reduced in areas that are characterized by the higher level of vegetation coverage. Considering the potential capacity of the endosymbiont for pathogen blocking, this discovery bears direct implications for public health. The potential role of *Wolbachia*, and to a lesser extent other endosymbionts, in pathogen transmission is being largely discussed nowadays (Garrigós et al., 2023). Alomar et al. approach the debate by comparing naturally *Wolbachia-*colonized *Cx. quinquefasciatus* mosquitoes to conspecific that were cured from this bacterium and aimed at understanding whether competition and *Wolbachia* infection in *Cx. quinquefasciatus* larvae have a combined effect on host fitness and vector competence to West Nile virus (WNV). Certainly, high competition stress affected different developmental traits, and increased susceptibility to WNV infection. However, *Wolbachia* infection displayed differential effects depending on the level of competition between larvae: under medium and high-competition, it enhances the survival of individuals to adulthood development and promote their survival, while in low-competition scenarios, the endosymbiont increases the susceptibility of adult mosquitoes to WNV. In sum, the interplay between native *Wolbachia* infection and elevated competition stress might yield unforeseen effects on the endosymbiont's ability to trigger pathogen blocking in nature, affecting its suitability for mosquito control interventions.

The role of mosquito microbiota have been further explored in this Research Topic in the review paper by Garrido et al. focus on the invasive Asian tiger mosquito, *Aedes albopictus*, one of the most invasive mosquito species in the world. Authors compiled and critically evaluated the updated literature on the interplay of mosquito microbiota and the transmission of *Ae. albopictus*-borne pathogens including Chikungunya, Dengue and Zika viruses. Given the potential threat posed by the expansion of *Ae. albopictus*, it is crucial to unravel the underlying mechanisms mediating the microbiota-disease risk interaction in both endemic and invaded areas where this species may create novel epidemiological scenarios.

While bacteria have been the most studied component of mosquito microbiota, other components are also relevant. This is the case of the mycobiome, which is the fungal component of the microbiome. Certain yeasts within the mycobiome can act as temporary niches for bacterial survival under stress, serving as reservoirs and alternative transmission routes. Additionally, some species colonizing the guts of insect vectors may interfere with pathogens. *Wickerhamomyces anomalus* is a yeast species that colonizes a broad diversity of habitats, including insect vectors. In this Research Topic, Cappelli et al. investigated the endobacterial communities of different *W. anomalus* strains that were isolated from various mosquito species known to transmit vector-borne diseases such as malaria and dengue. The authors revealed a ‘Matryoshka-like' association in the *Wa*F17.12 strain isolated from *Anopheles stephensi*, which is itself colonized by intravacuolar bacteria with antipathogenic properties against malaria parasites. These findings highlight the potential of nested interactions among yeasts, their endobacterial communities, and hosts, advancing our understanding of vectors biology.

The selection of hosts by insect vectors is a crucial stage in the transmission of vector-borne diseases (Martínez-De La Puente et al., 2021; Yan et al., 2021). Therefore, understanding the regulatory mechanisms involved in host-selection behavior is essential in suppressing pathogen transmission. Mosquitoes detect their bloodmeal sources using different cues, such as thermal, auditory, and chemical signals, i.e., anthropophilic species detect organic compounds relapsed from the bacterial skin of human hosts. However, the mechanisms that shape skin microbiome composition have not been fully understood. Bacterial communication by Quorum Sensing (QS) modulates their production of volatiles that may in turn affect host selection by mosquitoes. Kim et al. explored the potential of disrupting QS pathways as a novel control method to reduce mosquito attraction to a host. The results indicate that inhibiting the QS of the skin bacterium, *Staphylococcus epidermidis*, disrupts mosquito-microbiome communication. This reduces by half the attraction of adult *Aedes aegypti* to a blood meal.

Following a mosquito bite, pathogens are transferred into a new host through saliva, which itself may contain bacteria. Pathogens and bacteria may interact within the mosquito saliva and be simultaneously transferred to host tissues after a mosquito bite, but this pathway remains unexplored. Using an experimental approach, Accoti et al. documented the presence of *Serratia marcescens* bacteria in mosquito saliva as well as their ability to get transferred and efficiently colonize a mammalian host. This bacterium was also promoted by *Plasmodium berghei* and co-transmitted with it. Together, those results raise the possibility of mosquitoes serving as vectors of bacterial infection and highlight the potential utility of commensal mosquito bacteria for developing transmission-blocking strategies within a host.

In conclusion, the diverse array of studies within this Research Topic greatly enhances our understanding of the interactions among vectors, their microbiota, and the transmission of pathogens within the multifaceted framework of natural ecosystems and constitute a first step to enhance our understanding of pathogen epidemiology.

## Author contributions

MG: Writing—original draft, Writing—review & editing. GM: Writing—review & editing. JV: Writing—review & editing. JMP: Writing—original draft, Writing—review & editing.
